# Investigation of the Role of Genes Encoding Zinc Exporters *zntA*, *zitB*, and *fieF* during *Salmonella* Typhimurium Infection

**DOI:** 10.3389/fmicb.2017.02656

**Published:** 2018-01-11

**Authors:** Kaisong Huang, Dan Wang, Rikki F. Frederiksen, Christopher Rensing, John E. Olsen, Ana H. Fresno

**Affiliations:** ^1^Department of Veterinary and Animal Sciences, Faculty of Health and Medical Sciences, University of Copenhagen, Copenhagen, Denmark; ^2^National Key Laboratory of Agricultural Microbiology, College of Life Science and Technology, Huazhong Agricultural University, Wuhan, China; ^3^Institute of Environmental Microbiology, College of Resources and Environment, Fujian Agriculture and Forestry University, Fuzhou, China

**Keywords:** *S.* Typhimurium, zinc export, infection, amoebae, macrophages, mice

## Abstract

The transition metal zinc is involved in crucial biological processes in all living organisms and is essential for survival of *Salmonella* in the host. However, little is known about the role of genes encoding zinc efflux transporters during *Salmonella* infection. In this study, we constructed deletion mutants for genes encoding zinc exporters (*zntA*, *zitB*, and *fieF*) in the wild-type (WT) strain *Salmonella enterica* serovar Typhimurium (*S.* Typhimurium) 4/74. The mutants 4/74Δ*zntA* and 4/74Δ*zntA/zitB* exhibited a dramatic growth delay and abrogated growth ability, respectively, in Luria Bertani medium supplemented with 0.25 mM ZnCl_2_ or 1.5 mM CuSO_4_ compared to the WT strain. In order to investigate the role of genes encoding zinc exporters on survival of *S.* Typhimurium inside cells, amoeba and macrophage infection models were used. No significant differences in uptake or survival were detected for any of the mutants compared to the WT during infection of amoebae. In natural resistance-associated macrophage protein 1 (Nramp1)-negative J774.1 murine macrophages, significantly higher bacterial counts were observed for the mutant strains 4/74Δ*zntA* and 4/74Δ*zntA/zitB* compared to the WT at 4 h post-infection although the fold net replication was similar between all the strains. All four tested mutants (4/74Δ*zntA*, 4/74Δ*zitB*, 4/74Δ*fieF*, and 4/74Δ*zntA/zitB*) showed enhanced intracellular survival capacity within the modified Nramp1-positive murine RAW264.7 macrophages at 20 h post-infection. The fold net replication was also significantly higher for 4/74Δ*zntA*, 4/74Δ*zitB*, and 4/74Δ*zntA/zitB* mutants compared to the WT. Intriguingly, the ability to survive and cause infection was significantly impaired in all the three mutants tested (4/74Δ*zntA*, 4/74Δ*zitB*, and 4/74Δ*zntA/zitB*) in C3H/HeN mice, particularly the double mutant 4/74Δ*zntA/zitB* was severely attenuated compared to the WT in all the three organs analyzed. These findings suggest that these genes encoding zinc exporters, especially *zntA*, contribute to the resistance of *S.* Typhimurium to zinc and copper stresses during infection.

## Introduction

*Salmonella enterica* serovar Typhimurium (*S.* Typhimurium) is a major pathogen of animals and humans. In humans, the bacteria mainly cause foodborne salmonellosis; however, in immune-compromised patients, children, and the elderly, the bacteria may invade the host, survive within phagocytes, and cause a life-threatening systemic disease (Fields et al., 1986).

The ability of *Salmonella* to colonize specific niches impinges, among other factors, on the ability of the bacteria to obtain nutrients from the host, including transition metals. In this context, the relevance of iron acquisition during infection is well documented (Schaible and Kaufmann, 2004; Nairz et al., 2010). On the contrary, studies on the role of other metals, such as zinc, have emerged only in the last years (Ammendola et al., 2016).

Zinc, as other metal ions, is ubiquitously found in all organisms, where it plays a major structural and catalytic role in metalloenzymes, and has been reported to counter oxidative stress (Hood and Skaar, 2012). However, zinc is also toxic at high intracellular concentrations. Thus, the ability to sense zinc and to maintain cellular zinc homeostasis is essential for bacterial pathogens to be able to invade their hosts and cause disease (Porcheron et al., 2013). This is not only due to the need to ensure expression of enzymes related to bacterial metabolism, but also to ensure correct expression of virulence factors (Waldron and Robinson, 2009; Kehl-Fie and Skaar, 2010).

In order to fight infection, the host releases proteins that can chelate zinc and restrict its availability from invading pathogens (Diaz-Ochoa et al., 2014). Furthermore, the toxicity of zinc can be used as a host defense mechanism to promote bacterial killing. Thus, during infection, macrophages can target intracellular pathogens by modulating the intracellular concentrations of this metal ion (Kapetanovic et al., 2016). To counteract this, *Salmonella*, and other intracellular pathogens, have developed innovative mechanisms to maintain zinc homeostasis, enabling them to survive within macrophages (Behnsen et al., 2015; Kapetanovic et al., 2016). In *Escherichia coli*, not only zinc uptake systems, such as the high affinity transporters ZnuACB or ZinT (also known as YodA) but also zinc export systems, such as the P_1B_-type ATPase ZntA and the cation diffusion facilitators (CDFs) ZitB and YiiP, contribute to maintaining this homeostasis (Rensing et al., 1997; Grass et al., 2001; Wei and Fu, 2006).

In macrophages, natural resistance-associated macrophage protein 1 (Nramp1) is a transport system that sequesters divalent metal cations from the pathogen, slowing growth and preventing synthesis of enzymes (superoxide dismutases and catalases) for protection against the reactive oxygen species generated in response to infection in the bacterial cell, thus rendering the pathogen more susceptible to killing by oxidative stress (Govoni and Gros, 1998; Nelson, 1999).

Studies on the interplay between innate immunity and zinc during *S.* Typhimurium infection have so far been concerned with the role of zinc uptake during infection of the intestinal tract (Diaz-Ochoa et al., 2014). Recently, a study has shown how zinc trafficking works in *Salmonella* infected human macrophages (Kapetanovic et al., 2016) but the importance of such trafficking for the outcome of infection still needs to be determined. It has been further hypothesized that the ability to survive protozoan predation in the environment has contributed to the selection of copper/zinc resistance genes in bacteria (Hao et al., 2015; Chandrangsu et al., 2017). However, the role of genes involved in zinc export has never been studied during *Salmonella* infection of neither macrophages nor amoebae. Therefore, this work focused on how the lack of specific genes responsible for zinc export affected *S.* Typhimurium survival in amoebae and virulence. We show that zinc export is not important for survival in amoebae or in Nramp1-negative murine macrophages, while it did affect the way *S.* Typhimurium propagates inside Nramp1-positive murine macrophages and during infection of Nramp1-positive mice.

## Materials and Methods

### Strains and Construction of Mutants

All *Salmonella* strains tested in this study are listed in **Table 1**. The wild-type (WT) strain used for mutagenesis was *S.* Typhimurium 4/74 (Wallis et al., 1995). The derived mutants carrying deletions in *zntA*, *zitB*, *fief*, and *zntA/zitB* genes were obtained by lambda red-mediated homologous recombination as described (Datsenko and Wanner, 2000). Plasmids used for mutagenesis are also listed in **Table 1**.

**Table 1:** Strains and plasmids used in this work.

Strains/plasmids	Modification	Relevant features^∗^	Reference
Strain			
4/74	None	Wild-type. Virulent reference strain	Wallis et al., 1995
4/74Δ*zntA*	*zntA*::Apr	Defective in efflux of lead, cadmium, zinc, and mercury	This work
4/74Δ*zitB*	*zitB::*Apr	Defective in zinc efflux across the cytoplasmic membrane	This work
4/74Δ*zntA/zitB*	*zntA*::Apr *zitB*::Chl	Defective in transport of zinc, lead, cadmium, and mercury	This work
4/74Δ*fieF*	*fief*::Apr	Defective in a cation-efflux pump FieF induced by iron and zinc	This work
4/74Δ*ssaV*	*ssaV*::Kn	SPI2-T3SS defective	Unpublished
Plasmid			
pKD46	Ap	Plasmid with λ red recombinase expressed from arabinose inducible promoter	Doublet et al., 2008
pUO9090	Apr	Template used in lambda red PCR	Unpublished
pKD3	Chl	Template used in lambda red PCR	Datsenko and Wanner, 2000


*Ap, ampicillin; Apr, apramycin; Chl, chrolamphenicol; Kn, kanamycin. ^∗^According to the literature.*

### Media, Chemicals, and Growth Conditions

*Salmonella* was routinely grown in Luria Bertani (LB) medium (Oxoid, Denmark) at 37°C with aeration. Metal-supplemented conditions were achieved in LB supplemented with metals compounds; zinc (ZnCl_2_), copper (CuSO_4_), or manganese (MnCl_2_). Metals were prepared as 0.5 M stock solutions by solubilizing ultra-pure powders (Sigma, Denmark) in sterilized milliQ water.

Metals solutions were added to the media (final concentrations: 0.25 mM ZnCl_2_, 1.5 mM CuSO_4_, or 3 mM MnCl_2_) which were further inoculated with overnight cultures of the tested strains (final OD between 0.05 and 0.1) and the growth was assessed. OD was measured right after inoculation (T0) and at time 1.5, 3, 6, and 20 h post-inoculation. Non-supplemented LB was used as a control. The specific concentration of each metal compound tested in the growth assays above was based on results of a similar experiment, where the WT strain was grown in LB supplemented with increasing concentrations of the compound (0, 0.25, 0.5, 1, 1.5, 2, 3, 4, and 5 mM) and the growth was followed by measuring the OD at several time points. The maximum concentration of the metal allowing a normal growth of the WT (over which the compound was toxic to the strain) was selected for the final experiments. Antibiotics (Sigma) were used at the following concentrations: apramycin 75 mg/l, ampicillin 100 mg/l, chloramphenicol 30 mg/l, and kanamycin 50 mg/l when required.

### Whole-Genome Sequencing

Whole-genome sequencing of *S.* Typhimurium 4/74, and the derived isogenic strains: 4/74Δ*zntA*, 4/74Δ*zitB*, 4/74Δ*fieF*, and 4/74Δ*zntA/zitB*, was performed using the Illumina Genome Analyzer IIx (CD genomics, United States). Reads were assembled and contigs were aligned using the CLC Workbench Software (CLC Bio-Qiagen, Denmark) as previously described (Madoshi et al., 2016). Site-specific deletions in mutants were verified through comparative genomic analysis of the genomes of the mutated strains compared with the 4/74 genome sequence using the CLC software (Madoshi et al., 2016). Thus, the WGS approach allowed us to leave out laborious complementation experiments, as previously suggested (Bryant et al., 2012).

### Amoeba Infection Assays

The culture conditions for propagation of *Dictyostelium discoideum* strain AX4 (DBS0302402) and the protocol for infection studies were as previously described (Fey et al., 2007; Riquelme et al., 2016) with few modifications: Amoebae were maintained at 22°C in SM medium growing on a confluent lawn of *Klebsiella aerogenes* (DBS0305928). Before infection assays, amoebae were grown at 22°C in liquid HL5 medium (14 g/l tryptone, 7 g/l yeast extract, 0.35 g/l Na_2_HPO_4_, 1.2 g/l KH_2_PO_4_, 14 g/l glucose, pH 6.3) in the absence of bacteria (axenic cultures). When required, medium was supplemented with amikacin (100 mg/l) (Sigma).

Before infection, *D. discoideum* AX4 cells from mid-log cultures were collected by centrifugation (500 × *g*; 4 min). The supernatant was discarded and the pellet was washed once with HL5:DPBS (1:4 v/v) and resuspended in the same solution. Cells were counted in a hemocytometer (Fey et al., 2007) and adjusted to a concentration of 10^6^ cells/ml. Overnight cultures of the bacteria (WT 4/74 and its derived mutants) (**Table 1**) were harvested (12,000 × *g*; 2 min), and the pellets were washed once with HL5:DPBS and resuspended in the same solution. *D. discoideum* cells were co-incubated with the bacteria in 24-well polystyrene plates (MOI 100:1) at 22°C. Cells were also co-incubated with DPBS instead of bacteria (negative control). After 1 h of co-incubation, co-cultures were centrifuged (400 × *g*; 4 min) and washed once with HL5:DPBS. The infected cells were further incubated in HL5:DPBS supplemented with 100 mg/l amikacin at 22°C to kill extracellular bacteria. MIC for *S.* Typhimurium 4/74 and its isogenic mutants for amikacin were found to be <4 μg/ml. At 1 h post-infection (uptake; percentage of inoculated bacteria that are able to invade is estimated at this time point) the media supplemented with antibiotic was removed and the co-cultures were centrifuged at 400 × *g* for 4 min (*t* = 1 h), the pellet was washed once with DPBS and the infected amoebae were lysed with DPBS containing 0.2% Triton X-100 (v/v). The lysates were serially diluted and plated on LB agar plates. For the 6 h post-infection assay (*t* = 6 h) (survival assays) the media with antibiotic was also removed after 1 h and replaced by HL5:DPBS without antibiotic and left for another 5 h. At this time point, the amoebae were washed and lysed as previously mentioned and bacteria were enumerated. Fold net replication at *t* = 6 h was estimated with regards to *t* = 1 h post-infection (values for intra-cellular bacteria were expressed relatively to CFU detected for the specific strain at *t* = 1 h). The experiments were performed in quadruplicates.

### Infection of Murine Macrophages

Murine macrophage-like cells; J774.1 (Nramp1-negative) and an isogenic variant of RAW264.7 expressing Nramp1 (Nramp1-positive) (kind gift of G. Weiss) (Nairz et al., 2013) were used. Infection with WT 4/74 and isogenic strains (**Table 1**) was performed as previously described (Herrero-Fresno et al., 2014). The isogenic strain 4/74Δ*ssaV* was included as a negative control for the intracellular survival assays (**Table 1**). Briefly, macrophages were cultured in RPMI+GlutaMAX^TM^-I, Earles, 25 mM HEPES (Thermo Fisher Scientific, Denmark) supplemented with 10% (v/v) heat-inactivated FBS (Thermo Fisher Scientific) and 25 mg/l gentamycin (Sigma). The culture medium was supplemented with 0.01, 0.1, or 1 mM ZnCl_2_ in some experiments with Nramp1-positive macrophages. Cells were incubated in a humidified 37°C, 5% CO_2_ incubator, and phase-contrast microscopy was performed by using a Leica EL6000 microscope. For preparation of inocula, overnight cultures of the bacteria were diluted 1:100 and grown in LB to an exponential phase (OD = 0.6–0.8), harvested at 4000 × *g* for 5 min and resuspended in 0.9% (w/v) NaCl. CFU counts of the bacteria in the inoculum were verified by plating onto LB agar plates. Bacteria were added at a MOI of approximately 10:1. After 30 min of infection at 37°C, 5% CO_2_, the medium was removed and infected cells were washed twice with 0.9% NaCl. At this point (defined as time 0 h), fresh medium containing 250 mg/l amikacin was added to kill the extracellular bacteria. The macrophages were incubated for 1 h at 37°C, 5% CO_2_ in this solution, before new medium supplemented with 100 mg/l amikacin was added for the remaining part of the experiment. To perform CFU counts of the bacteria, cells were washed twice with 0.9% NaCl and lysed in 1 ml 0.1% Triton X-100 (v/v). The viable intracellular bacteria were enumerated by colony counts of lysate dilutions plated on LB agar plates. Bacteria were enumerated at *t* = 1 (uptake rate estimated here), *t* = 4 (survival 4 h post-infection), and *t* = 20 h (survival 20 h post-infection). Fold net replications at *t* = 4 and *t* = 20 h were estimated with regards to *t* = 1 h post-infection (values for intra-cellular bacteria at these time points were expressed relatively to CFU detected for the specific strain at *t* = 1 h). The assays were carried out in quadruplicates.

### Cytotoxicity Assays

Cytotoxicity toward macrophages was assessed as previously described (Herrero-Fresno et al., 2014). Briefly, at *t* = 20 h post-infection, the culture supernatants of infected macrophages were collected, transferred to 96-well plates, and cell production of cytosolic lactate dehydrogenase (LDH) was measured using the Colorimetric Cytotox 96 Kit (Promega, Denmark). Cytotoxicity was calculated as the percentage of LDH released in the supernatant of infected cells in relation to LDH released in the supernatant of non-infected, enzymically lysed cells (maximum release).

### Infection of Mice and Ethical Issues

This study was carried out in accordance with the principles expressed in the Declaration of Helsinki. Mice infection studies were performed with permission granted to John Elmerdahl Olsen from the Danish Animal Experiments Inspectorate, license number 2009/561-1675.

Three groups of six C3H/HeN mice each (4–5-week-old females) were infected intraperitoneally (i.p.) with the WT strain and one of the mutants 4/74Δ*zntA*, 4/74Δ*zitB*, or 4/74Δ*zntA/zitB* in a 1:1 ratio to determine the competitive index (CI) as described (Herrero-Fresno et al., 2014). Mutants and WT strain were grown overnight at 37°C with aeration, mixed at a 1:1 ratio, and diluted 1/100 in DPBS for inoculation. The exact concentration of bacteria in the inoculum was determined by serial dilution and plating onto LB agar. From this plate, 100 colonies were sub-cultured onto LB plates supplemented with apramycin (75 mg/l) to determine the ratio of WT to mutant strains. Mice were inoculated i.p. with a mixture of the mutant and WT strains in 100 μl of DPBS containing approximately 5 × 10^3^ CFU of each strain. Mice were euthanized at 5 days post-infection, and the spleens, livers, and mesenteric lymph nodes (MLN) were aseptically removed and homogenized. Each homogenate was serially diluted, and aliquots of the dilutions were plated onto LB plates. Hundred colonies collected from the LA plates were further spread on LB plates supplemented with apramycin to determine the exact ratio of WT and mutants recovered. CI values were calculated based on the ratio of the mutant/WT from the specific organ in relation to the mutant/WT ratio of the inoculum. A CI = 1 indicates that the virulence of the strains tested is equal. A CI < 1 shows that the mutant is less virulent than the WT.

### Statistical Analysis

The results are expressed as mean values ± standard deviations (SD) of at least three independent experiments. Statistical analysis was performed with the GraphPrism version 5.0 Software (GraphPad Inc.). Statistical significance was determined using one-way ANOVA with Dunnet’s multiple comparison post-test (amoebae and macrophage infections) or Tukey’s multiple comparison post-test (CIs from the mice infections). Statistical differences between WT and mutant strains in OD values (growth assays) and CFU counts (survival curves) were determined with one-sample *t*-test analysis. *P*-values < 0.05 were considered significant.

## Results

### Construction and Verification of Mutants Lacking Genes Encoding Zinc Exporters

To explore the role of zinc export in the interaction between *S.* Typhimurium and its host, the mutants: 4/74Δ*zntA*, 4/74Δ*zitB*, 4/74Δ*fieF*, and 4/74Δ*zntA/zitB* were constructed (**Table 1**). The WT isolate and the obtained mutants were subjected to whole-genome sequencing analysis. The comparative genome analysis showed that the mutant strains only differed from the WT strain by the site-specific deletions and insertion of an apramycin or chloramphenicol encoding cassette in the selected genes, indicating the successful construction of mutants for these genes.

### Growth Comparison between WT and Mutants in LB Medium and LB Medium Supplemented with Metal Compounds

**FIGURE 1:**
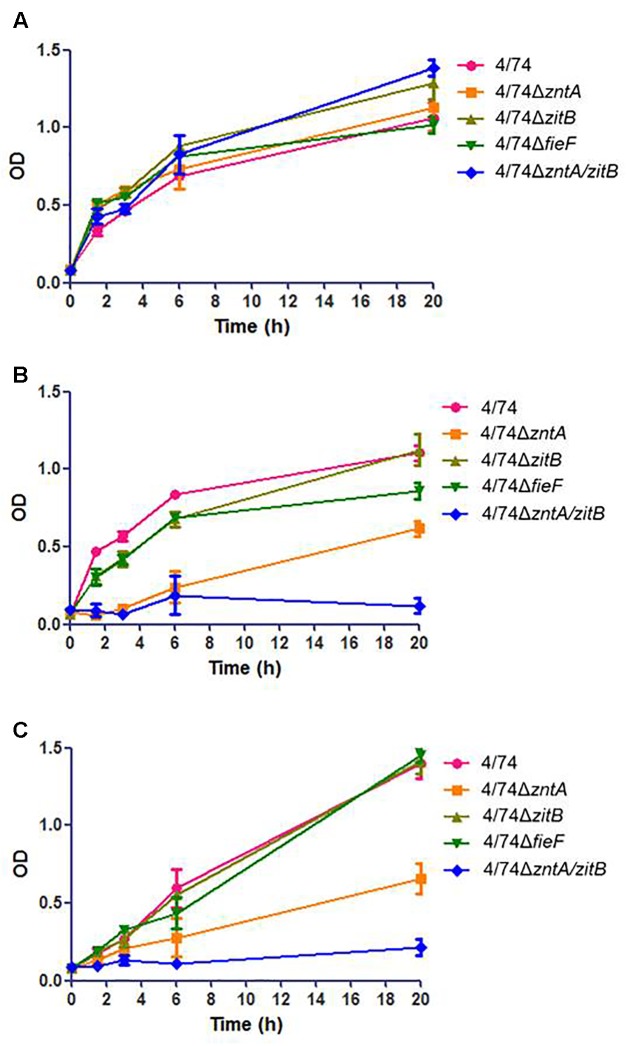
Analysis of the growth of the WT and zinc export mutants in: **(A)** LB. No statistically significant difference was observed between the WT and mutants. **(B)** LB supplemented with 0.25 mM ZnCl_2_. **(C)** LB supplemented with 1.5 mM CuSO_4_. When LB was supplemented with high concentrations of metals (ZnCl_2_ or CuSO_4_) the OD values for the mutants 4/74Δ*zntA* and 4/74Δ*zntA/zitB* were significantly lower than those observed for the WT at all the time points tested except T0 (*P*-value < 0.05).

To examine the role of the genes *zntA*, *zitB*, and *fieF* in trace metal trafficking in *S.* Typhimurium, the growth performances of the mutants and the WT strain in LB medium and LB medium supplemented, respectively, with 0.25 ZnCl_2_, 1.5 CuSO_4_ (**Figure 1**), or 3 mM MnCl_2_ (not shown) were compared. Concentrations above the ones listed for the growth assays resulted in severely attenuated growth of the WT strain (not shown).

All the mutants grew similarly to *S*. Typhimurium 4/74 (no statistically significant differences observed concerning ODs at any time point tested) in LB (**Figure 1A**). When 0.25 mM ZnCl_2_ was added to the medium, an obvious growth delay, with increased lag phase and with significantly lower OD values at all the time points tested except T0, was observed for the 4/74Δ*zntA* mutant, while the double mutant 4/74Δ*zntA/zitB* appeared to be unable to grow in LB supplemented with 0.25 mM ZnCl_2_ (**Figure 1B**). To examine whether these genes were also relevant for transport of other metals, growth was also analyzed in LB supplemented with 1.5 mM CuSO_4_ or 3 mM MnCl_2_. The 4/74Δ*zntA* mutant showed growth arrest and the 4/74Δ*zntA/zitB* mutant an almost abrogated growth when 1.5 mM CuSO_4_ was added to the media (**Figure 1C**) while addition of manganese did not affect the growth of any of the mutants (data not shown).

### Invasion and Survival Capability of the Zinc Export Deficient Mutants in Amoebae

**FIGURE 2:**
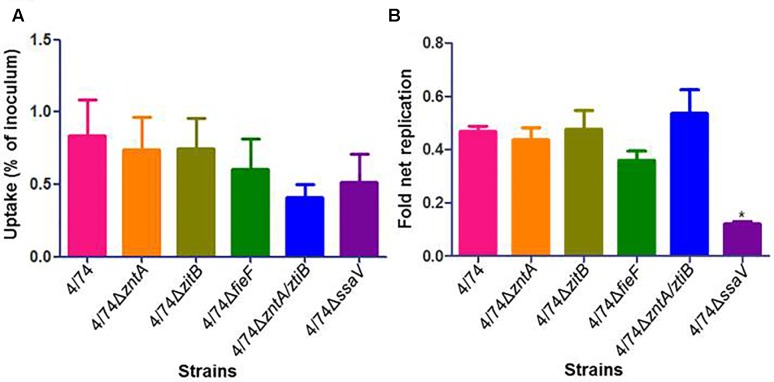
Infection of amoebae with WT and zinc export mutants. **(A)** Uptake by amoebae (percentage of inoculated bacteria that are able to infect). **(B)** Fold net replication at *t* = 6 versus *t* = 1 h. No significant differences were observed between the WT and the mutants neither in percentage of uptake nor in fold net replication at 6 h post-infection compared with 1 h post-infection.

Once the mutants deficient in zinc export were obtained and verified, we investigated their ability to infect and multiply within amoebae compared with the WT strain. Results showed no significant difference between isolates with respect to uptake by amoebae (**Figure 2A**) and no significant difference in their ability to replicate and survive inside these cells except for the control 4/74Δ*ssaV* which showed significantly lower replication rates than the WT as expected (**Figure 2B**). Even though there were no significant differences detected, the 4/74Δ*zntA/zitB* mutant seemed to show reduced uptake by amoebae compared with the WT (**Figure 2A**).

### Invasion and Survival Capability of the Zinc Export-Deficient Mutants in Macrophages

It has been well documented that the survival of *Salmonella* within macrophages is critical for causing systemic disease in the host (Fields et al., 1986; Alpuche-Aranda et al., 1995; Spano and Galan, 2012; Kurtz et al., 2017). Taking into account that Nramp1 acts as transport system of metal ions in macrophages, the role of the genes involved in zinc export during *S.* Typhimurium infection is likely to differ between macrophages expressing this transport system and macrophages not expressing Nramp1. Thus, we analyzed the invasion and survival ability of the zinc export-deficient mutants in both Nramp1-negative and Nramp1-positive murine macrophages.

**FIGURE 3:**
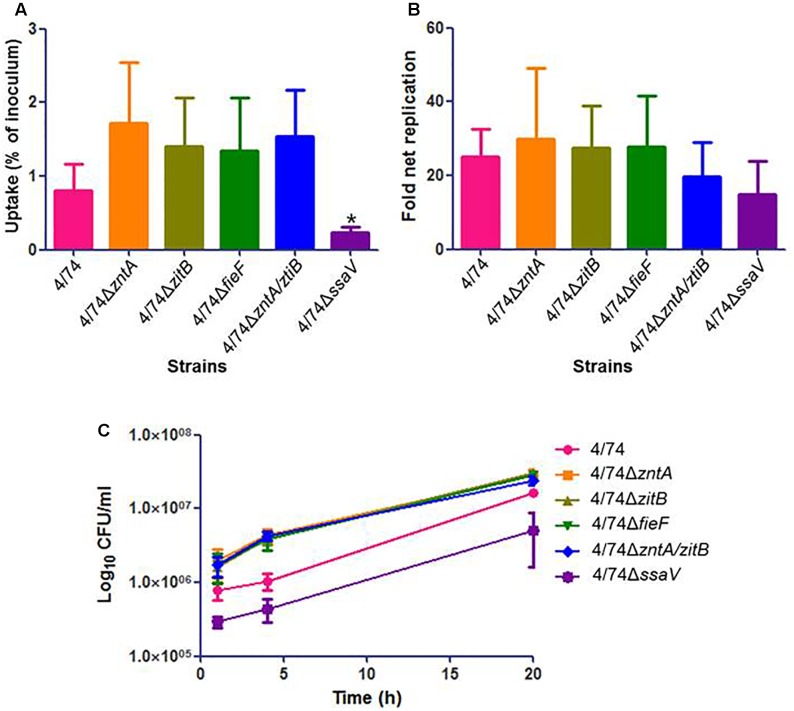
Infection of Nramp1-negative macrophages (J774.1) with the WT and zinc export mutants. **(A)** Uptake by macrophages (percentage of inoculated bacteria that are able to infect). **(B)** Fold net replication at *t* = 20 versus *t* = 1 h. **(C)** Survival curves. No significant differences were observed neither in the uptake by macrophages nor in fold net replications between the WT and mutants. At 4 h post-infection, the CFU counts for the mutants 4/74Δ*zntA* and 4/74Δ*zntA/zitB* were significantly higher than those detected for the WT (*P*-value < 0.05) although the fold net replications at this time point compared to *t* = 1 h were not significantly different between WT and mutants (not shown).

There were no statistically significant differences between the uptake of mutants and WT by the Nramp1-negative J774.1 macrophages (**Figure 3A**) while CFU counts for 4/74Δ*zntA* and 4/74Δ*zntA/zitB* were significantly higher than those obtained for the WT isolate at 4 h post-infection (**Figure 3C**). However, the fold net replication of none of the mutants was significantly different from that of the WT strain at this time point (not shown). At *t* = 20 h, both the CFU counts (**Figure 3C**) and the fold net replication (**Figure 3B**) were similar between all the strains tested.

**FIGURE 4:**
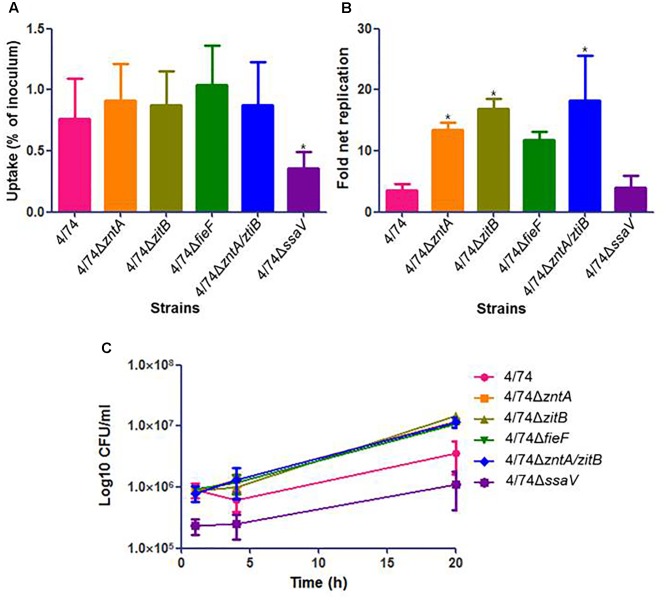
Infection of Nramp1-positive macrophages (RAW264.7) with WT and zinc export mutants. **(A)** Uptake by macrophages (percentage of inoculated bacteria that are able to infect). No significant differences were observed in the uptake by macrophages between WT and mutants. **(B)** Fold net replication at *t* = 20 versus *t* = 1 h. Fold net replications of 4/74Δ*zntA*, 4/74Δ*zitB*, and 4/74Δ*zntA/zitB* mutants were significantly higher than that of the WT 4/74 (*P*-value < 0.05). **(C)** Survival curves. At *t* = 20 h, all the mutants showed higher significant CFU counts than the WT (*P*-value < 0.05).

As shown for infection of Nramp1-negative macrophages, the uptake by Nramp1-positive macrophages did not differ significantly between strains (**Figure 4A**). Although no significant differences were observed concerning CFU counts (**Figure 4C**) and fold net replication (not shown), the trend was that all the mutants showed an increased survival rate compared to the WT at *t* = 4 h (**Figure 4C**). At *t* = 20 h, all four tested zinc export mutants led to significant higher CFU counts than the WT (**Figure 4C**). Also, all the four mutants showed higher fold net replication rates than the WT, although they were significantly higher only for 4/74Δ*zntA*, 4/74Δ*zitB*, and 4/74Δ*zntA/zitB* (**Figure 4B**). The addition of 0.01 mM ZnCl_2_ to the RMPI media led to similar trends in these results (not shown).

As expected, the previously described SPI2-defective mutant 4/74Δ*ssaV* (**Table 1**) exhibited an obviously reduced ability in invasion and survival within both kind of macrophages compared to the WT (**Figures 3C**, **4C**).

*S.* Typhimurium is highly cytotoxic to eukaryotic host cells, which has been widely reported in both epithelial and phagocytic cells (Santos et al., 2001; Cardenal-Munoz et al., 2014). In this study, the cytotoxic ability of the zinc export-deficient mutants toward macrophages was assessed and compared to the cytotoxicity levels generated by the WT on the basis of the amount of LDH released by macrophages at 20 h post-infection. The results showed no statistically significant different cytotoxic activity between the WT and the mutants in any of the cell lines tested. On the contrary, the levels of cytotoxicity of the control strain 4/74Δ*ssaV* were significantly lower than those detected for the WT in both cell lines (not shown).

### Analysis of the Role of Zinc Export during Systemic Infection of Mice

The co-infection of the Nramp1-positive mouse model with the WT 4/74 and each of the mutants: 4/74Δ*zntA*, 4/74Δ*zitB*, and 4/74Δ*zntA/zitB* showed that the zinc export gene *zntA* was required for systemic infection of mice. Thus, based on CFU counts obtained from spleen, liver, and MLN, the CIs of the mutant 4/74Δ*zntA* were significant lower than 1 (**Table 2**), indicating that the WT strain was able to outcompete this mutant when co-infecting mice. The 4/74Δ*zitB* mutant did not significantly differ from the WT based on CFU counts obtained from spleen and liver, while it was marginally, yet significantly, attenuated based on CFU counts obtained from MLN (CI significantly lower than 1) (**Table 2**). This gene, however, appeared to be important in the absence of *zntA*, since the double mutant 4/74Δ*zntA/zitB* was significantly more attenuated than 4/74Δ*zntA* in the spleen and the liver (**Table 2**).

**Table 2:** Competitive indices for *S.* Typhimurium WT 4/74 versus the derived mutants in mice.

*Salmonella* strains	Competitive index (CI)		
	
	Spleen	Liver	Mesenteric lymph nodes
*4/74*			
versus			
4/74Δ*zntA* (6)	0.35 ± 0.17^a^	0.37 ± 0.27^c^	0.24 ± 0.12^a^
4/74Δ*zitB* (6)	0.88 ± 0.25	1.05 ± 0.64	0.75 ± 0.21^c^
4/74Δ*zntA/zitB* (6)	0.07 ± 0.05^a,d^	0.09 ± 0.05^b,e^	0.13 ± 0.08^a^


*Competition indices were calculated based on input (CFU/ml of inoculum) and output (CFU/ml of organ sample) numbers of WT versus mutant bacteria as previously described (Herrero-Fresno et al., 2014). The results are shown as mean values based on the number of mice tested (indicated in brackets). All the six mice in each group survived to the end of the trial. ^a,b,c^CI was significantly different from 1.0: *P*-value < 0.0001, *P*-value < 0.001, and *P*-value < 0.05, respectively. ^d,e^CI was significnatly different from the mutant 4/74Δ*zntA: P*-value < 0.05 and *P*-value < 0.001, respectively.*

## Discussion

The metal ion zinc is the second most abundant transition metal in living organisms, playing a crucial catalytic or structural role in many vital biological processes such as gene expression, DNA replication, and general cellular metabolism. It also acts as cofactor for some virulence factors (Porcheron et al., 2013). On the other side, excess of zinc might lead to functional disorders and has been demonstrated to be deleterious to cells (Wang and Fierke, 2013; Chandrangsu et al., 2017). Therefore, a proper balance of the amount of zinc ions in all living organisms is essential, including pathogenic bacteria, which will encounter numerous different niches with different concentrations of this metal during the infection process. Several zinc uptake and export genes have been reported in *Enterobacteriaceae* encompassing *znuACB* and *zupT*, responsible for zinc uptake (Ammendola et al., 2007; Li et al., 2009; Cerasi et al., 2014) and *zntA*, *zitB*, and *yiiP* (also termed *fieF*) involved in export of zinc (Rensing et al., 1997; Grass et al., 2001; Wang et al., 2012). In this study, we report for the first time, the role of genes involved in zinc export in *S.* Typhimurium during infection of amoebae, murine macrophages, and mice.

We first investigated the role of zinc export-related genes: *zntA*, *zitB*, and *fieF* during *in vitro* growth in media supplemented with metal compounds at high concentrations. Results showed that when LB was supplemented with 0.25 mM ZnCl_2_, the mutants 4/74Δ*zitB* and 4/74Δ*fieF* exhibited a similar growth performance to that of the WT strain. However, a substantial attenuation and total restriction of growth were observed for the mutants 4/74Δ*zntA* and 4/74Δ*zntA/zitB*, respectively (**Figure 1B**), suggesting that the gene *zntA* plays a more important role than *zitB* in the export of zinc outside the cells and, thus enabling the survival of *S.* Typhimurium in the presence of high concentrations of zinc. This finding is consistent with a previous report on the role of the genes *zntA* and *zitB* in *E. coli*, which documented that *zitB*, that is constitutively expressed, only contributes to the maintenance of the zinc homeostasis under normal growth conditions or low zinc stress, while *zntA* is responsible for *E. coli* survival under high zinc concentrations conditions (Rensing et al., 1997; Outten and O’Halloran, 2001; Wang et al., 2012). It should be noted, however, that the growth restriction was more pronounced in the double mutant, suggesting that *zitB* might contribute to the export of zinc outside the cell under high zinc concentrations. The gene *fieF* has been shown not to be relevant for the survival of the bacteria under high concentrations of zinc either (Grass et al., 2001) which might explain our observations. Interestingly, when the medium was supplemented with 1.5 mM CuSO_4_, similar results to those observed when the strains were grown in the presence of ZnCl_2_ were observed (**Figure 1C**) suggesting that ZntA may also be involved in the export of copper. This is in agreement with previous studies in *E. coli*, where it was shown that ZntA is not only involved in the mobilization of zinc but also other divalent metal ions such as lead (Pb^2+^) and cadmium (Cd^2+^) (Binet and Poole, 2000). However, according to our results, manganese is not exported by this protein (not shown).

It has previously been shown that the zinc uptake system, *znuACB*, is required for zinc homeostasis when *Salmonella* grows in the intracellular milieu, and that the system contributes to *Salmonella* virulence (Campoy et al., 2002; Ammendola et al., 2007). On the contrary, little is known regarding the role of zinc export genes in *Salmonella* intracellular survival and virulence. We therefore tested the ability of the mutants lacking export zinc genes to invade and survive of/within mice macrophages compared to the WT. For this purpose, both Nramp1-negative and Nramp1-positive macrophages were used since the Nramp1 protein, expressed in the membrane of late phagosomes, was reported to be involved in the cellular distribution of divalent metals such as iron, zinc, and manganese, and strengthen and prolong the proinflammatory immune response (Fritsche et al., 2008; Hood and Skaar, 2012; Diaz-Ochoa et al., 2014).

In our study, all the four tested zinc export-related mutants exhibited enhanced intracellular survival abilities (significantly higher CFU counts than WT and higher fold net replications – significantly different from WT for 4/74Δ*zntA*, 4/74Δ*zitB*, and 4/74Δ*zntA/zitB*) within the Nramp1-positive murine macrophages at 20 h post-infection (**Figures 4B,C**). Thus, the lack of *fieF* appeared to not affect the survival rates compared to the WT as the deletion of the other export genes. These results suggest that Nramp1 is relevant to the growth of the WT and mutants under the conditions tested. According to previous research, the role of Nramp1 would be to pump divalent metal ions from the phagosome into the cytosol of the macrophage (Jabado et al., 2000; Forbes, 2003; Cellier et al., 2007). This might restrict the availability of metals required for the growth of *S.* Typhimurium. Thus, we speculate that the WT strain, where all the zinc export genes are present, actively exports zinc ions into the phagosome, which in turn are pumped out from the phagosome into the cytosol by Nramp1, thus limiting the growth ability of the bacteria. Besides, according to our observations and in agreement with previous observations in *E. coli* (Binet and Poole, 2000) not only zinc but other metals such as copper can be exported, and contribute to the growth phenotype in the Nramp1-expressing macrophages. The situation most likely does not represent the real infection situation (see mice model of infection below), where zinc and other metals from different sources reach the macrophages and could affect the intracellular growth of the pathogens. Since no zinc or other transition metals are present in the RPMI cell culture medium, this does not happen in the *in vitro* study.

Addition of 0.01 mM ZnCl_2_ to the cell culture medium led to similar results (not shown) suggesting that this amount of extracellular zinc did not change the zinc concentration dramatically in the phagosome within Nramp1-positive macrophages. Concentrations over 0.01 mM (0.1 and 1 mM) severely affected macrophages morphology, and their ability to form a monolayer (Supplementary Figure S1) and therefore could not be tested.

Several reports have proposed the overload of metals, mainly zinc and copper, as an immune strategy to restrict the pathogen growth within the host, and this hypothesis has been to some extent supported by a wide range of studies where the deletion of bacterial metal efflux-related systems leads to an impaired virulence of pathogens (Wagner et al., 2005; Ward et al., 2010; Botella et al., 2011). In *M. tuberculosis*, the deletion of the zinc export gene *ctpC*, belonging to the family of P_1B_-type ATPase exporters yielded substantially reduced growth ability within primary human macrophages in comparison with the WT strain (Botella et al., 2011). Intriguingly, these findings were opposite to our observations in *S.* Typhimurium, which were detailed above. Notably, the authors used human primary macrophages, where different intracellular concentrations of metals might be found compared to the mice tumor-associated macrophages used in this study.

It has been hypothesized that ability to withstand the toxic concentrations of zinc in mammalian phagocytes may have been acquired as a way to survive protozoan predation in the environment (Hao et al., 2015). Based on our results, the zinc export genes do not seem to be important for survival inside amoebae (**Figure 2**), despite them being Nramp1-positive cells. We cannot rule out that the results may have been different when using another model of protozoan, and further studies should be carried out before the hypothesis can be rejected.

Here, we also investigated the survival ability of the WT and the mutants within Nramp1-negative macrophages, and showed that in the absence of Nramp1, no phenotype could be associated to the zinc export genes (**Figure 3**). This is a good indication that zinc export is essential for growth inside the phagosome only when Nramp1 is present, since this is the place where Nramp1 is expressed. In the case of Nramp1-negative macrophages, apparently the lack of Nramp1 together with the lack of ion metals reaching the phagosomes do not lead to differences between WT and mutants deficient in zinc export genes and thus, zinc export might not be needed to survive under these conditions.

Since a phenotype was only observed in Nramp1-positive macrophages, we performed infection of Nramp1-positive mice. When co-infecting the animals with equal numbers of the WT and mutants lacking zinc export gene(s): 4/74Δ*zntA*, 4/74Δ*zitB* and 4/74Δ*zntA/zitB*, the 4/74Δ*zntA*, and 4/74*zntA/zitB* mutants were outcompeted by the WT. The mutant 4/74Δ*zitB* was slightly (but significantly) reduced in the MLN, but we concluded that the role of *zitB* in virulence was clearly less relevant compared to *zntA.* Thus, it appears that only WT bacteria, where both genes involved in metal mobilization are present, are able to fully maintain the metals ions homeostasis, and survive under these stressful conditions. These results are in disagreement with those obtained when infecting Nramp1-positive macrophages. This could be due to differences in metal ions availability and distribution between both scenarios. Thus, as mentioned above, *in vitro* and when Nramp1 is present, the absence of metals would lead to a situation where bacteria lacking metal export systems survive better than the WT since they can accumulate the required amount of metal ions needed for several physiological processes. If the export metal systems actively work, as expected for the WT, the bacteria might export and run out of intracellular metals needed for growth which would be in turn secreted out of the phagosomes by Nramp1. The situation might be totally different *in vivo*, where metal ions within macrophages (provided from different sources) may represent a stress factor for the bacteria which need to maintain a controlled balance of uptake, efflux, and accumulation of metal ions and to ensure that the metal availability is in accordance with their physiological needs. Thus, the lack of export systems might lead to an intracellular accumulation which might be toxic to the bacteria which would explain the reduced survival observed for the mutants compared to the WT. Additional studies are required for a clear elucidation of the observed disagreement.

In general, according to our results, we can conclude that zinc export plays a role in virulence of *S.* Typhimurium that might depend on the presence of Nramp1, availability of metals, and other aspects related to the infection model. Notably, among the three zinc export genes tested, *zntA* might be the most relevant to cope with zinc and copper stresses during *S.* Typhimurium infection.

## Author Contributions

CR conceived the study. KH, DW, RF, and AF performed the experiments. CR, JO, and AF participated in study design and provided critical advice. KH, DW, RF, JO, and AF analyzed the data, and AF wrote the first draft of the manuscript. All authors discussed the results and commented on the manuscript.

## Conflict of Interest Statement

The authors declare that the research was conducted in the absence of any commercial or financial relationships that could be construed as a potential conflict of interest.

## References

[B1] Alpuche-Aranda C. M. Berthiaume E. P. Mock B. Swanson J. A. Miller S. I. (1995). Spacious phagosome formation within mouse macrophages correlates with *Salmonella* serotype pathogenicity and host susceptibility. *Infect. Immun.* 63 4456–4462. 7591085 10.1128/iai.63.11.4456-4462.1995 PMC173634

[B2] Ammendola S. D’amico Y. Chirullo B. Drumo R. Civardelli D. Pasquali P. (2016). Zinc is required to ensure the expression of flagella and the ability to form biofilms in *Salmonella enterica* sv Typhimurium. *Metallomics* 8 1131–1140. 10.1039/C6MT00108D 27730246

[B3] Ammendola S. Pasquali P. Pistoia C. Petrucci P. Petrarca P. Rotilio G. (2007). High-affinity Zn^2+^ uptake system ZnuABC is required for bacterial zinc homeostasis in intracellular environments and contributes to the virulence of *Salmonella enterica*. *Infect. Immun.* 75 5867–5876. 10.1128/IAI.00559-07 17923515 PMC2168356

[B4] Behnsen J. Perez-Lopez A. Nuccio S. P. Raffatellu M. (2015). Exploiting host immunity: the *Salmonella* paradigm. *Trends Immunol.* 36 112–120. 10.1016/j.it.2014.12.003 25582038 PMC4323876

[B5] Binet M. R. Poole R. K. (2000). Cd(II), Pb(II) and Zn(II) ions regulate expression of the metal-transporting P-type ATPase ZntA in *Escherichia coli*. *FEBS Lett.* 473 67–70. 10.1016/S0014-5793(00)01509-X 10802061

[B6] Botella H. Peyron P. Levillain F. Poincloux R. Poquet Y. Brandli I. (2011). Mycobacterial p(1)-type ATPases mediate resistance to zinc poisoning in human macrophages. *Cell Host Microbe* 10 248–259. 10.1016/j.chom.2011.08.006 21925112 PMC3221041

[B7] Bryant J. Chewapreecha C. Bentley S. D. (2012). Developing insights into the mechanisms of evolution of bacterial pathogens from whole-genome sequences. *Future Microbiol.* 7 1283–1296. 10.2217/fmb.12.108 23075447 PMC3996552

[B8] Campoy S. Jara M. Busquets N. Perez De Rozas A. M. Badiola I. Barbe J. (2002). Role of the high-affinity zinc uptake *znuABC* system in *Salmonella enterica* serovar typhimurium virulence. *Infect. Immun.* 70 4721–4725. 10.1128/IAI.70.8.4721-4725.2002 12117991 PMC128140

[B9] Cardenal-Munoz E. Gutierrez G. Ramos-Morales F. (2014). Global impact of *Salmonella* type III secretion effector SteA on host cells. *Biochem. Biophys. Res. Commun.* 449 419–424. 10.1016/j.bbrc.2014.05.056 24858684

[B10] Cellier M. F. Courville P. Campion C. (2007). Nramp1 phagocyte intracellular metal withdrawal defense. *Microbes Infect.* 9 1662–1670. 10.1016/j.micinf.2007.09.006 18024118

[B11] Cerasi M. Liu J. Z. Ammendola S. Poe A. J. Petrarca P. Pesciaroli M. (2014). The ZupT transporter plays an important role in zinc homeostasis and contributes to *Salmonella enterica* virulence. *Metallomics* 6 845–853. 10.1039/c3mt00352c 24430377 PMC3969385

[B12] Chandrangsu P. Rensing C. Helmann J. D. (2017). Metal homeostasis and resistance in bacteria. *Nat. Rev. Microbiol.* 15 338–350. 10.1038/nrmicro.2017.15 28344348 PMC5963929

[B13] Datsenko K. A. Wanner B. L. (2000). One-step inactivation of chromosomal genes in *Escherichia coli* K-12 using PCR products. *Proc. Natl. Acad. Sci. U.S.A.* 97 6640–6645. 10.1073/pnas.120163297 10829079 PMC18686

[B14] Diaz-Ochoa V. E. Jellbauer S. Klaus S. Raffatellu M. (2014). Transition metal ions at the crossroads of mucosal immunity and microbial pathogenesis. *Front. Cell. Infect. Microbiol.* 4:2. 10.3389/fcimb.2014.00002 24478990 PMC3900919

[B15] Doublet B. Douard G. Targant H. Meunier D. Madec J. Y. Cloeckaert A. (2008). Antibiotic marker modifications of lambda Red and FLP helper plasmids, pKD46 and pCP20, for inactivation of chromosomal genes using PCR products in multidrug-resistant strains. *J. Microbiol. Methods* 75 359–361. 10.1016/j.mimet.2008.06.010 18619499

[B16] Fey P. Kowal A. S. Gaudet P. Pilcher K. E. Chisholm R. L. (2007). Protocols for growth and development of *Dictyostelium discoideum*. *Nat. Protoc.* 2 1307–1316. 10.1038/nprot.2007.178 17545967

[B17] Fields P. I. Swanson R. V. Haidaris C. G. Heffron F. (1986). Mutants of *Salmonella* typhimurium that cannot survive within the macrophage are avirulent. *Proc. Natl. Acad. Sci. U.S.A.* 83 5189–5193. 10.1073/pnas.83.14.5189 3523484 PMC323916

[B18] Forbes J. R. (2003). Iron, manganese, and cobalt transport by Nramp1 (Slc11a1) and Nramp2 (Slc11a2) expressed at the plasma membrane. *Blood* 102 1884–1892. 10.1182/blood-2003-02-0425 12750164

[B19] Fritsche G. Nairz M. Werner E. R. Barton H. C. Weiss G. (2008). Nramp1-functionality increases iNOS expression via repression of IL-10 formation. *Eur. J. Immunol.* 38 3060–3067. 10.1002/eji.200838449 18991287

[B20] Govoni G. Gros P. (1998). Macrophage NRAMP1 and its role in resistance to microbial infections. *Inflamm. Res.* 47 277–284. 10.1007/s000110050330 9719491

[B21] Grass G. Fan B. Rosen B. P. Franke S. Nies D. H. Rensing C. (2001). ZitB (YbgR), a member of the cation diffusion facilitator family, is an additional zinc transporter in *Escherichia coli*. *J. Bacteriol.* 183 4664–4667. 10.1128/JB.183.15.4664-4667.2001 11443104 PMC95364

[B22] Hao X. Luthje F. L. Qin Y. Mcdevitt S. F. Lutay N. Hobman J. L. (2015). Survival in amoeba–a major selection pressure on the presence of bacterial copper and zinc resistance determinants? Identification of a “copper pathogenicity island”. *Appl. Microbiol. Biotechnol.* 99 5817–5824. 10.1007/s00253-015-6749-0 26088177

[B23] Herrero-Fresno A. Wallrodt I. Leekitcharoenphon P. Olsen J. E. Aarestrup F. M. Hendriksen R. S. (2014). The role of the *st313-td* gene in virulence of *Salmonella* Typhimurium ST313. *PLOS ONE* 9:e84566. 10.1371/journal.pone.0084566 24404174 PMC3880295

[B24] Hood M. I. Skaar E. P. (2012). Nutritional immunity: transition metals at the pathogen-host interface. *Nat. Rev. Microbiol.* 10 525–537. 10.1038/nrmicro2836 22796883 PMC3875331

[B25] Jabado N. Jankowski A. Dougaparsad S. Picard V. Grinstein S. Gros P. (2000). Natural resistance to intracellular infections: natural resistance-associated macrophage protein 1 (Nramp1) functions as a pH-dependent manganese transporter at the phagosomal membrane. *J. Exp. Med.* 192 1237–1248. 10.1084/jem.192.9.1237 11067873 PMC2193348

[B26] Kapetanovic R. Bokil N. J. Achard M. E. Ong C. L. Peters K. M. Stocks C. J. (2016). *Salmonella* employs multiple mechanisms to subvert the TLR-inducible zinc-mediated antimicrobial response of human macrophages. *FASEB J.* 30 1901–1912. 10.1096/fj.201500061 26839376

[B27] Kehl-Fie T. E. Skaar E. P. (2010). Nutritional immunity beyond iron: a role for manganese and zinc. *Curr. Opin. Chem. Biol.* 14 218–224. 10.1016/j.cbpa.2009.11.008 20015678 PMC2847644

[B28] Kurtz J. R. Goggins J. A. Mclachlan J. B. (2017). *Salmonella* infection: interplay between the bacteria and host immune system. *Immunol. Lett.* 190 42–50. 10.1016/j.imlet.2017.07.006 28720334 PMC5918639

[B29] Li Y. Qiu Y. Gao H. Guo Z. Han Y. Song Y. (2009). Characterization of Zur-dependent genes and direct Zur targets in *Yersinia pestis*. *BMC Microbiol.* 9:128. 10.1186/1471-2180-9-128 19552825 PMC2706843

[B30] Madoshi B. P. Kudirkiene E. Mtambo M. M. Muhairwa A. P. Lupindu A. M. Olsen J. E. (2016). Characterisation of commensal *Escherichia coli* isolated from apparently healthy cattle and their attendants in Tanzania. *PLOS ONE* 11:e0168160. 10.1371/journal.pone.0168160 27977751 PMC5158034

[B31] Nairz M. Schleicher U. Schroll A. Sonnweber T. Theurl I. Ludwiczek S. (2013). Nitric oxide-mediated regulation of ferroportin-1 controls macrophage iron homeostasis and immune function in *Salmonella* infection. *J. Exp. Med.* 210 855–873. 10.1084/jem.20121946 23630227 PMC3646493

[B32] Nairz M. Schroll A. Sonnweber T. Weiss G. (2010). The struggle for iron - a metal at the host-pathogen interface. *Cell. Microbiol.* 12 1691–1702. 10.1111/j.1462-5822.2010.01529.x 20964797

[B33] Nelson N. (1999). Metal ion transporters and homeostasis. *EMBO J.* 18 4361–4371. 10.1093/emboj/18.16.4361 10449402 PMC1171511

[B34] Outten C. E. O’Halloran T. V. (2001). Femtomolar sensitivity of metalloregulatory proteins controlling zinc homeostasis. *Science* 292 2488–2492. 10.1126/science.1060331 11397910

[B35] Porcheron G. Garenaux A. Proulx J. Sabri M. Dozois C. M. (2013). Iron, copper, zinc, and manganese transport and regulation in pathogenic *Enterobacteria*: correlations between strains, site of infection and the relative importance of the different metal transport systems for virulence. *Front. Cell. Infect. Microbiol.* 3:90. 10.3389/fcimb.2013.00090 24367764 PMC3852070

[B36] Rensing C. Mitra B. Rosen B. P. (1997). The *zntA* gene of *Escherichia coli* encodes a Zn(II)-translocating P-type ATPase. *Proc. Natl. Acad. Sci. U.S.A.* 94 14326–14331. 10.1073/pnas.94.26.14326 9405611 PMC24962

[B37] Riquelme S. Varas M. Valenzuela C. Velozo P. Chahin N. Aguilera P. (2016). Relevant genes linked to virulence are required for *Salmonella* Typhimurium to survive intracellularly in the social amoeba *Dictyostelium discoideum*. *Front. Microbiol.* 7:1305. 10.3389/fmicb.2016.01305 27602025 PMC4993766

[B38] Santos R. L. Tsolis R. M. Baumler A. J. Smith R. III Adams L. G. (2001). *Salmonella enterica* serovar typhimurium induces cell death in bovine monocyte-derived macrophages by early *sipB*-dependent and delayed *sipB*-independent mechanisms. *Infect. Immun.* 69 2293–2301. 10.1128/IAI.69.4.2293-2301.2001 11254586 PMC98158

[B39] Schaible U. E. Kaufmann S. H. (2004). Iron and microbial infection. *Nat. Rev. Microbiol.* 2 946–953. 10.1038/nrmicro1046 15550940

[B40] Spano S. Galan J. E. (2012). A Rab32-dependent pathway contributes to *Salmonella* Typhi host restriction. *Science* 338 960–963. 10.1126/science.1229224 23162001 PMC3693731

[B41] Wagner D. Maser J. Lai B. Cai Z. Barry C. E. III. Honer (2005). Elemental analysis of *Mycobacterium avium-, Mycobacterium tuberculosis*-, and *Mycobacterium smegmatis*-containing phagosomes indicates pathogen-induced microenvironments within the host cell’s endosomal system. *J. Immunol.* 174 1491–1500. 10.4049/jimmunol.174.3.1491 15661908

[B42] Waldron K. J. Robinson N. J. (2009). How do bacterial cells ensure that metalloproteins get the correct metal? *Nat. Rev. Microbiol.* 7 25–35. 10.1038/nrmicro2057 19079350

[B43] Wallis T. S. Paulin S. M. Plested J. S. Watson P. R. Jones P. W. (1995). The *Salmonella dublin* virulence plasmid mediates systemic but not enteric phases of salmonellosis in cattle. *Infect. Immun.* 63 2755–2761. 7790094 10.1128/iai.63.7.2755-2761.1995 PMC173368

[B44] Wang D. Fierke C. A. (2013). The BaeSR regulon is involved in defense against zinc toxicity in *E. coli*. *Metallomics* 5 372–383. 10.1039/c3mt20217h 23446818 PMC3657296

[B45] Wang D. Hosteen O. Fierke C. A. (2012). ZntR-mediated transcription of *zntA* responds to nanomolar intracellular free zinc. *J. Inorg. Biochem.* 111 173–181. 10.1016/j.jinorgbio.2012.02.008 22459916 PMC3408962

[B46] Ward S. K. Abomoelak B. Hoye E. A. Steinberg H. Talaat A. M. (2010). CtpV: a putative copper exporter required for full virulence of *Mycobacterium tuberculosis*. *Mol. Microbiol.* 77 1096–1110. 10.1111/j.1365-2958.2010.07273.x 20624225 PMC2965804

[B47] Wei Y. Fu D. (2006). Binding and transport of metal ions at the dimer interface of the *Escherichia coli* metal transporter YiiP. *J. Biol. Chem.* 281 23492–23502. 10.1074/jbc.M602254200 16790427

