# Supplementary figures and images for: Investigation of the Role of Genes Encoding Zinc Exporters zntA, zitB, and fieF during Salmonella Typhimurium Infection
Source: Front Microbiol. 2018 Jan 11;8:2656. doi: 10.3389/fmicb.2017.02656 (PMC5768658; doi:10.3389/fmicb.2017.02656)

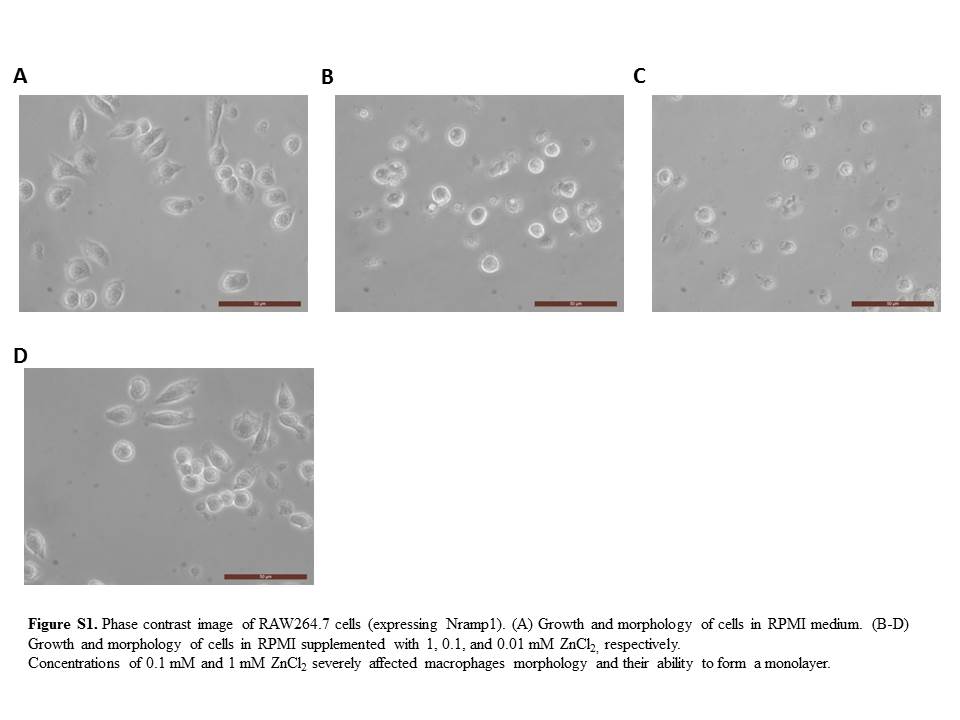

Supplement: Supplementary file 1 [file Image_1.JPEG]
